# The First Description of Dominance Hierarchy in Captive Giraffe: Not Loose and Egalitarian, but Clear and Linear

**DOI:** 10.1371/journal.pone.0124570

**Published:** 2015-05-13

**Authors:** Edita Horová, Karolína Brandlová, Markéta Gloneková

**Affiliations:** Department of Animal Science and Food Processing in Tropics and Subtropics, Czech University of Life Sciences Prague, Prague, Czech Republic; University of Tasmania, AUSTRALIA

## Abstract

Wild giraffes live in extensive groups in the fission fusion system, maintaining long social distances and loose social bonds. Within these groups, resources are widely distributed, agonistic encounters are scarce and the dominance hierarchy was reported in males only, while never deeply analysed. In captivity, the possibility to maintain inter-individual distances is limited and part of the resources is not evenly distributed. Consequently, we suggest that agonistic encounters should be more frequent, leading to the establishment of the dominance hierarchy. Based on the differences in resource-holding potential, we suggested that the rank of an individual would be affected by age and sex. Based on hypotheses of prior ownership, we tested whether rank was positively affected by the time spent in a herd and whether it was stable in adult females, which were present long-term in the same herd. We originally monitored four herds of Rothschild giraffes (*Giraffa camelopardalis rothschildii*) in Dvůr Králové zoo (n = 8), Liberec zoo (n = 6), and two herds in Prague zoo: Prague 1 (n = 8) and Prague 2 (n = 9). The Prague 1 and Prague 2 herds were then combined and the resulting fifth herd was observed over three consecutive years (2009, 2010, and 2011) (n = 14, 13, and 14, respectively). We revealed a significantly linear hierarchy in Dvůr Králové, Prague 2 and in the combined herd in Prague. Rank was significantly affected by age in all herds; older individuals dominated the younger ones. In females, rank was positively affected by the time spent in the herd and adult females in Prague maintained their rank during three consecutive years. This study represents the first analysis of the dominance hierarchy in the captive giraffe, and discusses the behavioural flexibility of the social structure in response to monopolisable resources in a captive environment.

## Introduction

Life in a group for prey species often evolved as a response to predation pressure [1]. Benefits as increased vigilance, foraging efficiency, and better offspring survival force individuals together, in balance with costs that arise from close proximity. The costs of group living are mostly connected with competition, either for mates or resources [1]. Due to this competition, individuals often participate in agonistic interactions. When these interactions are easily identified and repeated in a consistent way, they can be referred to as dominance—subordinate interactions based on a winner and loser effect [2]. These interactions may lead to the establishment of dominance hierarchies that decrease the overall level of agonistic interactions within the group [1], since the subordinate individuals might actively avoid conflict with the dominant ones. The formation of such interactions presumes that an individual can recognise and remember other group members [3]. An individual’s rank is mostly used as a measure of its position in the hierarchy [4–6]. Both agonistic ranks based on the interactions and competitive feeding ranks based on differential access to food can be measured and tend to be strongly correlated [7]. The rank orders are mostly sex- and age-dependent [8] and correlate with many behavioural variables, such as reactions to novelty and learning performance [9], and success at foraging and reproduction [10–15]. The dominance rank order however, might reflect the resource-holding potential (RHP) of an individual [16,17]. The resource holding potential is based on the assessment of rival’s abilities and probability of the conflict escalation over the resource or withdrawal from the resource. The higher is the difference between rivals, the lower is probability of conflict escalation. Therefore, an individual assesses its rival according to external features (as size or body-mass) and decides whether to enter the conflict or not [17]. Alternatively, the experience of an individual may play role in the hierarchy formation, based on prior ownership. Despite of lower body mass, an individual may be in possession of a resource and develops an effective strategy for its protection. In such a case, the rival approaching a resource may lose despite of larger size or higher body-mass [18,19]. Those two approaches are not mutually exclusive. Once formed, the rank orders can be stable for long periods or continually reassessed. The stability of female social rank over time should provide important benefits as increased reproductive success [20].

Species that live in loose groups with abundant and widespread resources are not expected to establish dominance hierarchies to gain priority access to such an “unlimited” resource [21]. Moreover, interactions that lead to the establishment and maintenance of hierarchies can be stressful to subordinate animals, and might also result in serious or fatal injuries [12,22], suggesting that the hierarchies are formed when such costs are outweighed by benefits gained from priority access to resources. Abundant and widespread resources are typical for the most of wild ungulates, but not for captive ones. In captivity, two types of food are usually provided—forage and concentrates. While the access to the forage is generally unlimited, the concentrates are fed in limited amount per head, and they are generally considered attractive resource. The feeding rank in ungulates is therefore often studied during feeding of concentrates [23–26]. Limited resources in wild giraffes cause changes in females and also males social behaviour [27], while even water may be considered such a limited resource [28], we can therefore expect such a behavioural change also in captive environment.

In the captive environment an individual is unable to leave the group to avoid the interaction, but is forced to stay in the group and deal with its herdmates. Such artificial change of conditions may lead to the expression of behavioural flexibility of the species in the changing environmental conditions [29], corresponding to the presumptions that in the limited space of stables and outdoor enclosures, animals establish a mechanism to prevent inter-individual conflicts over limited resources as the concentrate feeding is [3,30,31]. The ability of a species to exhibit behavioural flexibility to environmental conditions was proved to be important for its survival [29], provides advantages in new environments [30] and during habitat alterations [31].

The giraffe (*Giraffa camelopardalis*) is an example of a prey species with loose social bonds that uses abundant and widespread resources. Social bonds among giraffes have been recently discussed and perceptions of the social system have switched from loose aggregations of individuals with non-lasting social bonds [32–34] to an advanced fission—fusion system with an elaborated communication structure [35–38].

Wild giraffes live in low densities and rarely approach conspecifics, except for feeding at the same tree and maintaining a distance over 20 m apart [32]. Social interactions in wild giraffes are very subtle and are restricted mainly to mother—offspring contact and the agonistic encounters of males [39]. The social life of wild female giraffes has been described as an association of small groups of a few members, which generally includes calves and occasionally some younger males [36,40,41]. Groups are temporary and their size depends on the season [41]. Temporary changes in group size were first explained by Bercovitch and Berry [42], who described giraffe social structure as a fission—fusion system within large groups. This also corresponds with evidence that wild female giraffes form stable populations within an area [40]. The dominance hierarchy has been described for male giraffes only [34] and has not been deeply analysed. The theory that the long neck of a giraffe evolved to gain dominance in sexual encounters [43] has recently been abandoned [44,45].

The Rothschild giraffe (*Giraffa camelopardalis rothschildi*) belongs to the Red List of endangered taxa [46] about which limited knowledge from the wild exists. Rothschild giraffes inhabit dense savannah woodland in Uganda and Kenya with fewer than 1,100 wild individuals remaining. They are successfully bred in captivity mainly in European and US zoos, with numbers exceeding 500 living individuals (according to ZIMS—Zoological Information Management System 2014), thus providing an excellent base for research and conservation.

A recent publication of Bashaw et al. [47] demonstrated that Rothschild giraffes in captivity have a complex social structure. This fact challenges the original opinion that the only strong bonds among giraffes are between a mother and her dependent young [48]. Furthermore, the experimental social separation of captive Rothschild giraffes provided another evidence of complex and long-term relationships of giraffe [49]. Another finding of Bashaw [50] supports the fact that captive giraffes maintain social relationships and suggests that studies of giraffe social relationships and activity are applicable across a range of captivity conditions [51]. However, the dominance hierarchy has never been studied in captive giraffes.

Given that the possibility to maintain social distances in captivity similar to those in the wild is limited by the size of enclosure or stable, the number of social encounters increases. Moreover, the access to preferred food (pellets, vegetables or concentrates) is limited to several occasions throughout the day, resulting in unequally distributed resources. Because of this, we suggest that the benefits of hierarchy formation (priority access to resources) outweigh the costs (risk of injury during agonistic encounters) and (i) the dominance hierarchy will form in captive giraffes. Based on the difference in resource holding potential [16], we presume that the rank of an individual will be affected (ii) by age and (iii) by sex. Captive herds of giraffes are often stable, with unrelated individuals joining the herd only due to breeding management. We presumed that (iv) rank would be positively affected by the time spent in a herd, regarding the asymmetry of prior ownership [18,19] and (v) that rank would be stable among adult females in the periods when no adults enter or leave the herd.

## Materials and Methods

### Ethic Statement

The observations of giraffes in zoos took place in most cases from the visitors' area. To the breeding facilities the observer came only when it was necessary. The observer did not disturb the animals, did not influence their behaviour or interfere with the daily management in the stables. Behavioural sampling did not affect the animals in any manner. Observations were approved by head zoologists responsible for the animals in each zoo, namely: Jaroslav Šimek, Prague Zoo, Luboš Melichar, Liberec Zoo, and Luděk Čulík, Dvůr Králové Zoo. No other specific permissions were required.

### Study Sites and Subjects

Data were collected in captive Rothschild giraffe herds in three zoological gardens in the Czech Republic, namely in Prague Zoo between 2008 and 2011, in Dvůr Králové Zoo in 2010, and in Liberec Zoo in 2010. All observed individuals were born in captivity, as well as their parents. The observations were performed from the visitors′ area or from the keepers′ area when necessary. The observations did not influence the behaviour of the studied animals and observers did not alter the daily routine procedures of husbandry in any zoo.

The zoo enclosures differed in size (Prague Zoo—400 m^2^ indoor, 2.2 ha outdoor, Liberec Zoo 700 m^2^ indoor, 0.14 ha outdoor, Dvůr Králové Zoo—270 m^2^ indoor, 0.22 ha outdoor), but were similar in structure. Each outside enclosure was formed of a sandy surface with grass and several trees. Inside enclosures were littered with sawdust. All herds were fed by forage *ad libitum*, formed by hay or grass accompanied by branches for browsing. Concentrated feed was provided in the form of grain fodder, fresh fruit and vegetables, which was provided twice-daily and consumed immediately. Access to water was provided *ad libitum*.

We originally monitored four herds of Rothschild giraffes (*Giraffa camelopardalis rothschildii*) in Dvůr Králové Zoo (eight individuals), Liberec Zoo (six individuals), and two herds in Prague Zoo: Prague 1 (eight individuals) and Prague 2 (nine individuals). The Prague 1 and Prague 2 herds were then combined and the resulting fifth herd was observed in three consecutive years (2009, 2010, and 2011) as Joined 1 (14 individuals), Joined 2 (13 individuals) and Joined 3 (14 individuals). Adults, sub adults and juveniles were present in all observed herds (S1–S7 Tables). Giraffes have naturally distinctive markings [39] and therefore, all studied animals were identified individually by their coat pattern, body size, shape of the horns, shape of the hooves, and sex.

### Data Collection

Data collection occurred during daytime hours. The total time of observation was 240 h in all zoos (Table 1). Agonistic encounters with a clear submissive reaction were recorded into a loss and win table *ad libitum* [52], during feeding. Win and loss table contained information about the identity of each individual, date of the observation, time of the beginning and end of the observation period, who was the winner of the encounter, who was the recipient, what kind of threat it was. We recorded the most apparent forms of aggression, including necking, strikes with the head also described as bumping [53,54], as well as milder forms of aggression, including threats, pushes and chases. Pushes and chases occurred when the aggressor moved directly towards the loser in a line to intercept it, then either remained in the site formerly occupied by the loser, or continued moving through the point of intersection whilst the loser moved away (a chase). Seeber et al. [53] called chase yield, Bashaw [55] called it avoiding. Threat was described as unspecified aggression, Seeber et al. [53] refers to it as displace and Bashaw [55] summarised it as non-contact aggression. Only agonistic interactions were recorded, in which the loser apparently avoided the winner, adopted a submissive posture and did not return to the conflict, as only these interactions demonstrate that the submissive individual accepted its subordinate position. Observations were made until filling the loss and win table.

**Table 1:** Results of linearity in all studied herds.

Locality	Season	Number of individuals	Number of interactions	Observed hours	Landau's index (h)	Corrected index (h')	*P* value	Hierarchy
Dvůr Králové	2010	8	111	13	0.73	0.74	*P* < 0.05	Linear
Liberec	2010	6	51	10	0.69	0.73	*P* > 0.05	Near-linear
Praha 1	2008	8	118	65	0.56	0.64	*P* = 0.08	Near-linear
Praha 2	2008	9	156	67	0.69	0.73	*P* < 0.05	Linear
Joined 1	2009	14	339	35	0.52	0.56	*P* < 0.05	Linear
Joined 2	2010	13	313	33	0.48	0.53	*P* < 0.05	Linear
Joined 3	2011	14	265	17	0.82	0.84	*P* < 0.05	Linear

### Data Analyses

To determine whether (i) a hierarchy existed in the captive giraffe herds, we created dominance matrices. These matrices originated from loss and win tables, which were filled in during observations. For each agonistic encounter, the winner and loser were recorded, allowing the calculations of wins and losses for each individual. Loss and win tables were analysed in MatMan software and were compiled by the I&SI method [5]. The aim of the I&SI method is to identify an order that is the most consistent with a linear hierarchy. Firstly, it minimises the number of inconsistencies (dyads for which the actual dominance relationship does not correspond with the relationship in the hierarchy found). Secondly, it minimises the total strength of the inconsistencies *SI*, subject to the condition that *I* is at its minimum [56]. We evaluated the transitivity of dominance relationships among group members, based on submissive behaviours. We used de Vries′ improved version of Landau′s index of linearity [57], correcting for unknown and tied relationships (h′). The value of h′ varies from 0, indicating absence of linearity, to 1, indicating complete linearity [58,59]. A value h′ higher than 0.80 was taken to indicate a strongly linear hierarchy [59].

To test whether (ii) older individuals have a higher rank than younger individuals, (iii) males are higher in rank than females and (iv) individuals that spent more time in a herd are on a higher rank, we calculated Clutton-Brock Index (CBI) [60], as it provides detailed information not only about the order within hierarchy, but also about the relative distances between individuals. The CBI for each member, i, of a group was calculated using the formula described in detail by Gammell et al. [61]: *CBI*(i) = (*B* + ∑*b* + 1) / (*L* + ∑*l* + 1), where *B* represents the number of individuals that *i* defeated in one or more interactions, ∑*b* represents the total number of individuals (excluding *i*) that those represented in *B* defeated, *L* represents the number of individuals by which *i* was defeated and ∑*l* represents the total number of individuals (excluding *i*) by which those represented in *L* were defeated [61–63]. An individual′s rank range from 1, which is the top rank, and it further goes up to the number of individuals in a group. We also used the CBI rank for comparison of the changes among three consecutive years in the combined herd in Prague.

As the data were not normally distributed, we used logarithmic transformation of CBI values. Transformed CBI values showed normal distribution (Kolmogorov-Smirnov test, d = 0.14, p > 0.2). All analyses were performed in software STATISTICA 2011 version 9.1. For predictions (ii, iii) we analysed the influence of age and sex on the CBI values using General Linear Model (GLM), with “age” (years) as continuous variable and “sex” (male, female) as categorical variable. We also tested the interaction of “age” and “sex” within the model.

As age and time spent in the herd were correlated, for analysis of (iv) time spent in the herd we used linear regression and we also tested the differences between sexes.

## Results

In 240 total hours of observation time, 1,353 agonistic interactions were observed between individuals. The dominance matrices of herds in Dvůr Králové and Prague 2 revealed a significant linear hierarchy (i). The dominance matrices of the combined herd Prague revealed a significant linear hierarchy for all three consecutive years. The dominance matrix of the Liberec and Prague 1 herds were near-linear (Table 1).

**Fig 1:**
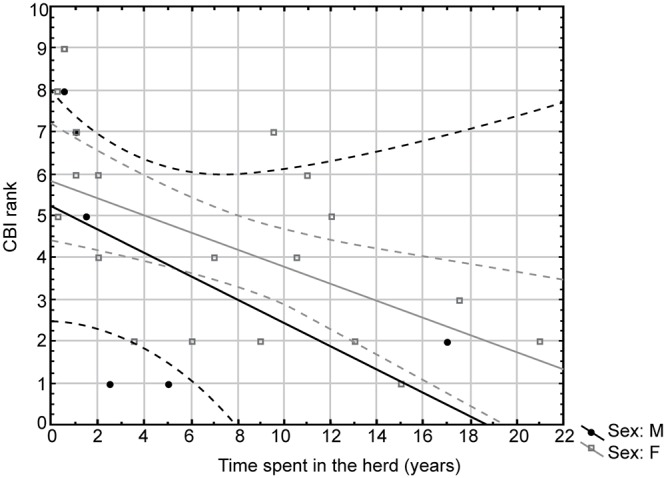
Rank vs. age. The CBI rank was significantly (F_(1, 5.168)_ = 13.16, *P* < 0.001) affected by age. Note that the highest rank level is 1 (the first position in a hierarchy).

**Fig 2:**
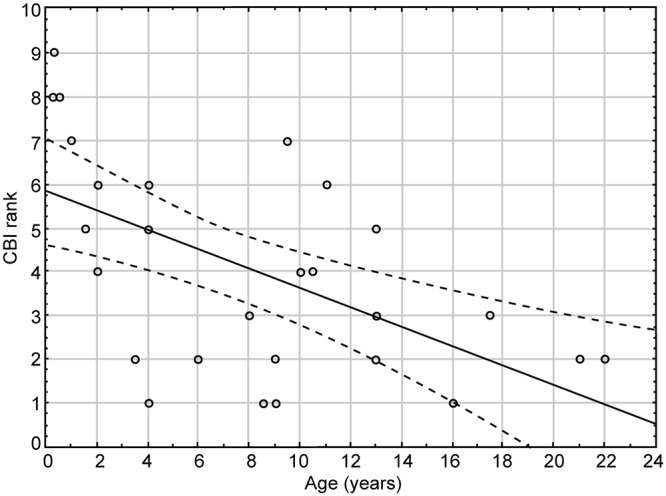
Rank vs. time in a herd. The CBI rank was influenced by time spent in the herd in females only (r^2^ = 0.25, p = 0.017), not in males (r^2^ = 0.35, p = 0.09). Note that the highest rank level is 1 (the first position in a hierarchy).

We confirmed that (ii) CBI values for individual giraffes were significantly (F _(1, 5.168)_ = 13.16, *P* < 0.001) affected by age (Fig 1). (iii) Sex did not affect the CBI of mixed giraffe herds neither separately (F_(1, 0.007)_ = 0.01, p = 0.89) nor in interaction with age (F_(1, 0.553)_ = 1.4, p = 0.25). (iv) CBI was positively affected by the time spent in the herd (r^2^ = 0.02, *P* < 0.05) (Fig 2). Further analyse showed that CBI was influenced by time spent in the herd in females only (r^2^ = 0.25, p = 0.017), not in males (r^2^ = 0.35, p = 0.09).

**Fig 3:**
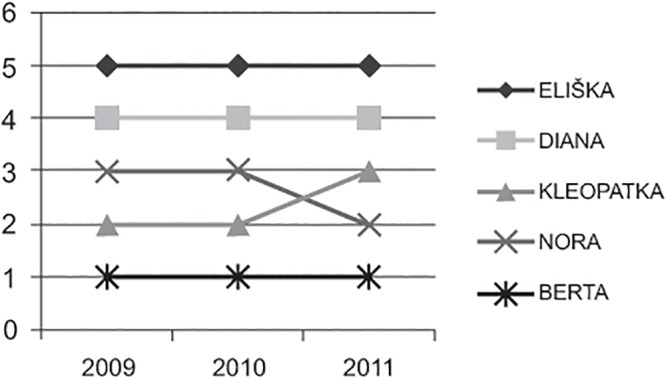
Rank stability. Changes in the CBI rank of five adult females during three consecutive years in the combined herd in Prague.

(v) Adult females in Prague (herds Joined 1, Joined 2, and Joined 3) maintained their rank during three consecutive years with only one exception of two females that exchanged their positions relative each another (Fig 3).

## Discussion

This study represents the first detailed analysis of the dominance hierarchy in the captive giraffe. The dominance hierarchy in wild giraffes has only been reported in bulls, dividing them into three age classes [64]. Nevertheless, studies that focused on the fission—fusion system in giraffes stated that the hierarchy did not exist in giraffes [37] or the studies did not consider the hierarchy or dominance relationships at all [35,36,65]. Neither the studies of social relationships in captive giraffes considered dominance relationship and hierarchy formation [47,49]. However, the dominance hierarchy as a part of social environment may substantially contribute to the formation of social system, together with generally represented ecological factors, and influence the association patterns [66].

Formation of hierarchy in captive Rothschild giraffes corresponds to the presumptions that in the limited space of stables and outdoor enclosures, giraffes establish a mechanism to prevent inter-individual conflicts over limited resources [3,67,68]. Hierarchy formation might help captive giraffes to save energy and prevent the risk of injuries during frequent interactions [3]. The dominant giraffes gained priority access to food, and had the possibility to select preferred food components as shown by Ceacero et al. [69]. Nevertheless, most of the captive ungulates (both wild and domestic) face similar restriction in space and limited access to concentrated food, which is not common in their natural environment. Despite this, not all of them develop dominance hierarchies in captivity, e.g. camels (KB—unpublished data). The formation of hierarchy in the captive giraffe therefore appears to reflect the behavioural flexibility of the species facing unnatural captive conditions.

The hierarchical structures in observed captive giraffe herds were mostly significantly linear or had high values of linearity indices even when not significant. This is not always the case in ungulate societies, where triangular or complex dominance relationships are common [57,70,71]. The linearity of hierarchies was stronger in larger giraffe herds (8–14 individuals) than in smaller ones (6–8 individuals), in contrary to the findings of Favre et al. [15], where linearity decreased with the increasing size of a herd. Linear hierarchies have been often reported in female-bonded ungulate groups [58,72], which are formed by closely tied and related females. The linearity of the dominance hierarchy in the giraffe supports the findings of recent studies showing that the relationships among female giraffes are stronger than previously thought [47,51].

Similarly to other ungulates, the position in the hierarchy was strongly affected by the age of an individual. In captive Rothschild giraffes, adult individuals dominated subadults and juveniles and the subadults dominated juveniles. Older individuals often occupy the top hierarchical positions in ungulate groups [11,73–75]. Although age is strongly correlated with body mass before individuals reach adulthood, in adult animals, age might have a higher impact than body mass [76]. An age- (and/or body mass) based linear hierarchy was found in other savannah species living in a fission—fusion system, where the resources are abundant, but some scarce patchily distributed resources favoured the establishment of such a hierarchy [58].

We observed no difference between the rank of males and females of the same age class. We observed herds with mixed sexes in all cases, but there was always only one adult male in the herd due to management and safety reasons. It is therefore likely that the lack of effect is a function of the small sample size in case of males. Nonetheless, the rank of the adult male was the highest of all individuals except for one case, even though males were neither the tallest nor the oldest individuals. The mean ranks of juvenile males tended to be higher than that of juvenile females, probably due to the differences in growing patterns and resulting sexual dimorphism [33]. Given the sample size of our studied group, we suggest that sex and/or size might play a role in achievement of the dominance rank, as in Correa et al. [72].

We also suggested that the rank was positively correlated with the time spent in a herd, although there was a confounding effect of age, as the number of individuals joining the herd during adulthood was not sufficient for the detail statistical evaluation. The time spent in a herd was related to the rank of females but not to the rank of males, which further support the existence of stronger bonds among females in the fission fusion system. This finding corresponds with the asymmetries connected with prior ownership [18]. Hierarchies might be based on such asymmetries and respected, even if they do not correspond to the differences in RHP, such as body mass [19]. This might also reflect the fact that older females or females that live for a longer time in an area are better able to maintain their dominance because of experience.

Once established, the hierarchy among adult females appears to be stable, as shown in the combined herd in Prague. All females maintained their ranks during three consecutive years, with one exception: two females with a position in the hierarchy very close to one another exchanged their ranks during the final year of observations. We cannot derive any general conclusion from this case, as this was the only herd that was observed in three consecutive seasons. Nevertheless, this result corresponds very well with a similar situation for other captive ungulates [11,22].

Hierarchy formation in captive giraffes appears to be based on their RHP, although we did not obtain data for body mass or other phenotypic traits that enabled certain individuals to acquire dominance over individuals that are unable to oppose them. However, before adulthood, age in giraffe is strongly correlated with size [77]. After reaching adult size, age becomes independent of physical traits such as body mass or height in giraffes [77]. Age-driven rank position might then lead to hierarchy formation based on mutual benefits from avoiding the conflicts over patchily distributed usurpable resources [58].

The evidence for hierarchy in captive giraffes suggests the existence of behavioural flexibility in an evolutionary old species. Wild giraffes are non-territorial animals [32,78] and rely on abundant, widely distributed resources [79–81], thus, their social relationships might remain unresolved because there is no need to dominate over others in terms of feeding competition [58]. However, although individuals are not constantly together in the fission—fusion system, clear dominance hierarchies among society members might be formed, to dominate over patchily distributed resources [7,58,66]. Given the decrease in suitable habitats for giraffes and the differential nutritional value of plants in the savannah ecosystem [82], the feeding preferences of individual giraffes might lead to the existence of competitive feeding behaviour in the future, leading to hierarchy formation as shown in the example of captive giraffes. The existence of dominance hierarchies might therefore influence the association patterns and social structure of the giraffe herds [66,83,84]. This pilot study of the hierarchy in captive giraffe herds demonstrates a behavioural change as a response to modified living conditions and suggests the direction for studies in broader sociobiological context.

## Conclusions

Although the relationships among giraffes are often described as loose and subtle, we demonstrated that a clear linear dominance hierarchy existed in captive giraffe herds. The rank of an individual was affected by its age and the rank of females was stable during subsequent observational seasons. The establishment hierarchy was based on the resource-holding potential over limited resources in captivity. The characteristics of hierarchy reflected those found in other female-bonded ungulates and the formation of a linear hierarchy in captivity reflected the behavioural flexibility of the giraffe in facing different environmental conditions.

## Supporting Information

S1 Table Composition of herd Prague 1. (DOCX)

S2 Table Composition of herd Prague 2. (DOCX)

S3 Table Composition of herd Dvůr Králové. (DOCX)

S4 Table Composition of herd Liberec. (DOCX)

S5 Table Composition of herd Joined1. (DOCX)

S6 Table Composition of herd Joined2. (DOCX)

S7 Table Composition of herd Joined3. (DOCX)
