# Supplementary material for: The First Description of Dominance Hierarchy in Captive Giraffe: Not Loose and Egalitarian, but Clear and Linear
Source: PLoS One. 2015 May 13;10(5):e0124570. doi: 10.1371/journal.pone.0124570 (PMC4430478; doi:10.1371/journal.pone.0124570)
Supplement: S1 Table — (DOCX) [file pone.0124570.s001.docx]

Tab. 1: Composition of herd Praha 1.

| Herd Praha 1 | | | | | |
| --- | --- | --- | --- | --- | --- |
| Name | Date of Birth | Age (years) | Sex | Category | Rank according CBI |
| Kleopatra | 13.1.1993 | 16 | F | AD | 1 |
| Šimon | 21.12.1986 | 22 | M | AD | 2 |
| Kasunga | 2.7.2000 | 8 | F | AD | 3 |
| Hana | 16.8.2006 | 2 | F | SUB | 4 |
| Luděk | 26.8.2007 | 1.5 | M | SUB | 5 |
| Václav | 28.9.2007 | 1.5 | M | SUB | 5 |
| Bořek | 11.7.2008 | 1 | M | JUV | 7 |
| Gabriela | 3.3.2009 | 0.25 | F | JUV | 8 |
