# Supplementary material for: The First Description of Dominance Hierarchy in Captive Giraffe: Not Loose and Egalitarian, but Clear and Linear
Source: PLoS One. 2015 May 13;10(5):e0124570. doi: 10.1371/journal.pone.0124570 (PMC4430478; doi:10.1371/journal.pone.0124570)
Supplement: S2 Table — (DOCX) [file pone.0124570.s002.docx]

Tab. 2: Composition of herd Praha 2.

| Herd Praha 2 | | | | | |
| --- | --- | --- | --- | --- | --- |
| Name | Date of Birth | Age (years) | Sex | Category | Rank according CBI |
| Johan | 20.12.1999 | 9 | M | AD | 1 |
| Berta | 25.3.1988 | 21 | F | AD | 2 |
| Eliška | 6.10.1995 | 13 | F | AD | 2 |
| Nora | 27.6.1999 | 9 | F | AD | 2 |
| Diana | 6.1.2003 | 6 | F | AD | 2 |
| Nikola | 28.11.1997 | 11 | F | AD | 6 |
| Mahulena | 17.11.2007 | 1 | F | SUB | 7 |
| Slávek | 19.1.2009 | 0.5 | M | JUV | 8 |
| Bedřiška | 1.3.2009 | 0.25 | F | JUV | 9 |
