# Supplementary material for: The First Description of Dominance Hierarchy in Captive Giraffe: Not Loose and Egalitarian, but Clear and Linear
Source: PLoS One. 2015 May 13;10(5):e0124570. doi: 10.1371/journal.pone.0124570 (PMC4430478; doi:10.1371/journal.pone.0124570)
Supplement: S3 Table — (DOCX) [file pone.0124570.s003.docx]

Tab. 3: Composition of herd Dvůr Králové.

| Herd Dvůr Králové | | | | | |
| --- | --- | --- | --- | --- | --- |
| Name | Date of Birth | Age (years) | Sex | Category | Rank according CBI |
| Tommy | 2.3.2002 | 8.5 | M | AD | 1 |
| Jenifer | 11.2.2007 | 3.5 | F | AD | 2 |
| Nina | 12.7.1997 | 13 | F | AD | 3 |
| Kenia | 2.5.2000 | 10 | F | AD | 4 |
| Jaruna | 2.7.1997 | 13 | F | AD | 5 |
| Johari | 5.10.2006 | 4 | F | AD | 6 |
| Etola | 20.11.2001 | 9,5 | F | AD | 7 |
| Ozák | 5.1.2010 | 0.5 | M | JUV | 8 |
