# Supplementary material for: The First Description of Dominance Hierarchy in Captive Giraffe: Not Loose and Egalitarian, but Clear and Linear
Source: PLoS One. 2015 May 13;10(5):e0124570. doi: 10.1371/journal.pone.0124570 (PMC4430478; doi:10.1371/journal.pone.0124570)
Supplement: S4 Table — (DOCX) [file pone.0124570.s004.docx]

Tab. 4: Composition of herd Liberec.

| Herd Liberec | | | | | |
| --- | --- | --- | --- | --- | --- |
| Name | Date of Birth | Age (years) | Sex | Category | Rank according CBI |
| Twiga | 9.12.2006 | 3.5 | F | AD | 1 |
| Miky | 24.8.2006 | 4 | M | AD | 2 |
| Nancy | 30.3.1993 | 17.5 | F | AD | 3 |
| Sandra | 27.1.2000 | 10.5 | F | AD | 4 |
| Vanesa | 28.7.2006 | 4 | F | AD | 5 |
| Nela | 21.4.2008 | 2 | F | SUB | 6 |
