# Supplementary material for: The First Description of Dominance Hierarchy in Captive Giraffe: Not Loose and Egalitarian, but Clear and Linear
Source: PLoS One. 2015 May 13;10(5):e0124570. doi: 10.1371/journal.pone.0124570 (PMC4430478; doi:10.1371/journal.pone.0124570)
Supplement: S5 Table — (DOCX) [file pone.0124570.s005.docx]

Tab. 5: Composition of herd Joined 1.

| Herd Joined 1 | | | | | |
| --- | --- | --- | --- | --- | --- |
| Name | Date of Birth | Age (years) | Sex | Category | Rank according CBI |
| Johan | 20.12.1999 | 10 | M | AD | 1 |
| Berta | 25.3.1988 | 22 | F | AD | 2 |
| Kleopatra | 13.1.1993 | 17 | F | AD | 3 |
| Nora | 27.6.1999 | 10 | F | AD | 4 |
| Bořek | 11.7.2008 | 2 | M | SUB | 5 |
| Faraa | 30.10.2007 | 2.5 | F | SUB | 6 |
| Nikola | 28.11.1997 | 12 | F | AD | 7 |
| Diana | 6.1.2003 | 7 | F | AD | 8 |
| Eliška | 6.10.1995 | 14 | F | AD | 9 |
| Jiří | 28.4.2009 | 0.75 | M | JUV | 10 |
| Bedřiška | 1.3.2009 | 0.75 | F | JUV | 11 |
| Slávek | 19.1.2009 | 1 | M | JUV | 12 |
| Gabriela | 8.3.2009 | 0.75 | F | JUV | 13 |
| Laura | 30.5.2009 | 0.5 | F | JUV | 14 |
